# Mapping thermal conductivity across bamboo cell walls with scanning thermal microscopy

**DOI:** 10.1038/s41598-019-53079-4

**Published:** 2019-11-13

**Authors:** Darshil U. Shah, Johannes Konnerth, Michael H. Ramage, Claudia Gusenbauer

**Affiliations:** 1 Centre for Natural Material Innovation, Dept. of Architecture, University of Cambridge, Cambridge, CB2 1PX UK; 2 Institute of Wood Technology and Renewable Materials, Department of Material Sciences and Process Engineering, University of Natural Resources and Life Sciences Vienna, Konrad-Lorenz-Strasse 24, 3430 Tulln an der Donau, Austria

**Keywords:** Plant sciences, Composites, Biomaterials

## Abstract

Scanning thermal microscopy is a powerful tool for investigating biological materials and structures like bamboo and its cell walls. Alongside nanoscale topographical information, the technique reveals local variations in thermal conductivity of this elegant natural material. We observe that at the tissue scale, fibre cells in the scattered vascular tissue would offer preferential pathways for heat transport due to their higher conductivities in both anatomical directions, in comparison to parenchymatic cells in ground tissue. In addition, the transverse orientation offers more resistance to heat flow. Furthermore, we observe each fibre cell to compose of up to ten layers, with alternating thick and thin lamellae in the secondary wall. Notably, we find the thin lamellae to have relatively lower conductivity than the thick lamellae in the fibre direction. This is due to the distinct orientation of cellulose microfibrils within the cell wall layers, and that cellulose microfibrils are highly anisotropic and have higher conductivity along their lengths. Microfibrils in the thick lamellae are oriented almost parallel to the fibre cell axis, while microfibrils in the thin lamellae are oriented almost perpendicular to the cell axis. Bamboo grasses have evolved to rapidly deposit this combination of thick and thin layers, like a polymer composite laminate or cross-laminated timber, for combination of axial and transverse stiffness and strength. However, this architecture is found to have interesting implications on thermal transport in bamboo, which is relevant for the application of engineered bamboo in buildings. We further conclude that scanning thermal microscopy may be a useful technique in plant science research, including for phenotyping studies.

## Introduction

Engineered bamboo is an exciting family of materials that has attracted much interest in applications for sustainable construction^1–3^. Today, products such as laminated bamboo are most commonly used as flooring materials due to their hardness and durability. However, their stiffness and strength is comparable to engineered wood products, making them suitable for structural uses as well^2,3^. For applications in buildings, thermal properties of materials are also relevant. Thermal conductivity, for instance, dictates the rate of temperature increase through a material, which affects fire behaviour and building energy performance. Energy use in buildings (e.g. space heating and cooling) accounts for over 30% of global energy consumption and CO_2_ emissions^4–6^. In this regard, material choices and their thermal performance have a notable role in improving building energy intensities^4–6^.

**Figure 1:**
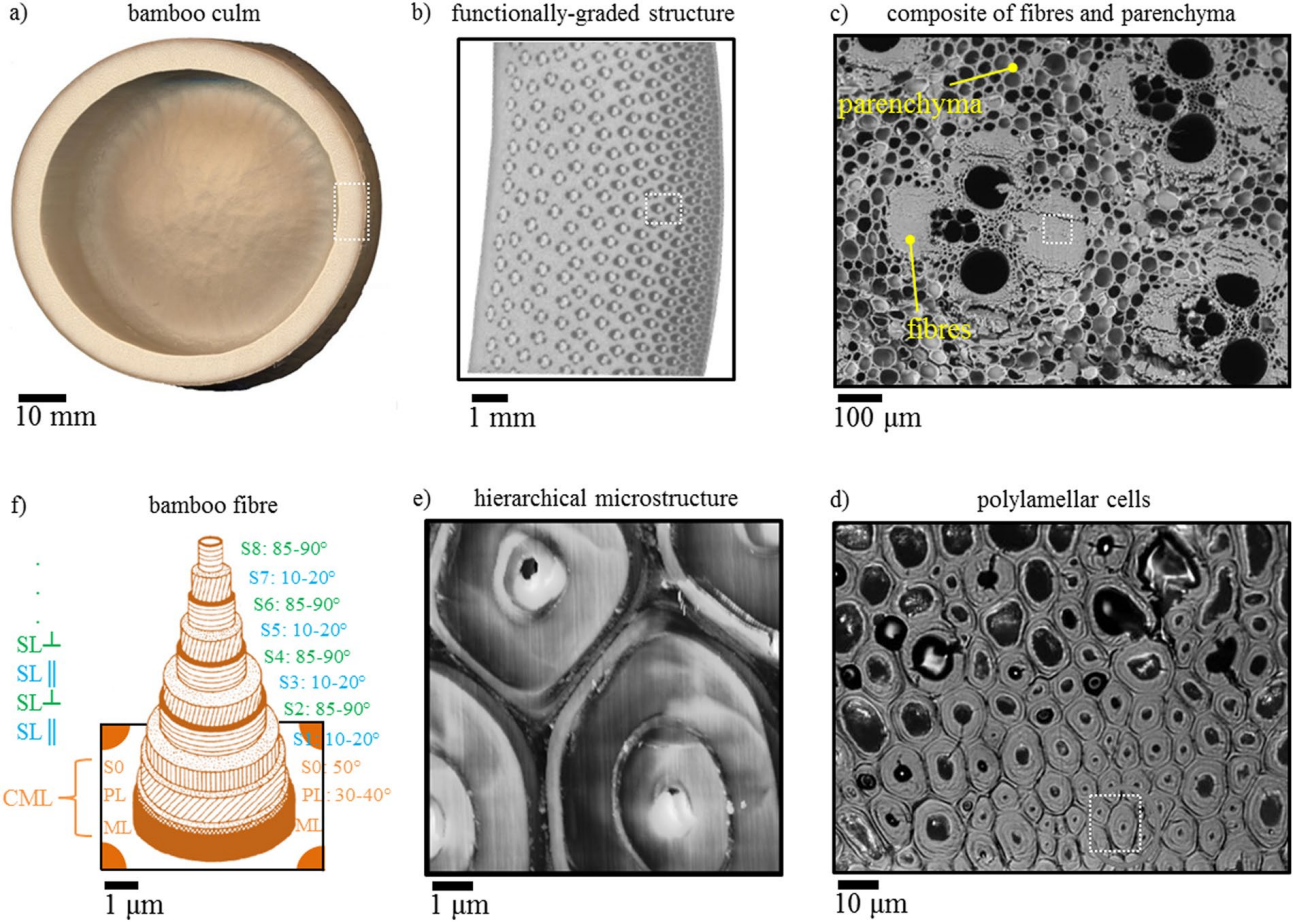
Schematic of bamboo’s hierarchical ultrastructure. (**a**) Bamboo is a monocot grass, typically characterised by a hollow, segmented culm. (**b**) The culm wall is functionally-graded, with an increasing density of stiff fibre-comprising vascular bundles towards the epidermis. (**c**) These vascular bundles are embedded in ground tissue of box-shaped parenchyma cells with thin primary cell walls (PL). (**d**–**f**) The fibres have thick lignified cell walls and a polylamellar structure, which includes a primary cell wall layer, and as many as eight secondary cell wall layers (SL). A pectin-rich middle lamella (ML) adjoins fibre cells together. As it can be difficult to distinguish the ML and PL from each other, the compound middle lamella (CML) refers to the combination of ML, PL and the first layer of the secondary cell wall (S0) of each fibre cell.

While the ultrastructure of bamboo^7–9^ and its relation to mechanical properties (mainly stiffness)^10–12^ are well-known, the thermal behaviour of bamboo, particularly in relation to its structure, is only sparsely reported in literature^13,14^. In previous work^13^, experiments at the macro-scale (i.e. on samples that were 10–20 mm thick, >50 mm diameter) have established that thermal conductivity of bamboo is a structure-dependent property. Specifically, volumetric composition, reflected by the apparent density, has a well-predicted effect on thermal transport properties of bamboo. Based on semi-empirical composite models, the study was also able to estimate that the thermal conductivity of the bamboo cell wall material is *k*_║_ = 0.55–0.59 W/m·K in the longitudinal direction (along the culm length), and *k*_⊥_ = 0.39–0.43 W/m·K in the transverse/radial direction. However, these single characteristic conductivity values do not reflect the heterogeneity in bamboo cell types (e.g. in ground and vascular tissue) and bamboo’s complex, hierarchical, lamellar structure (Fig. 1). Information on thermal conductivity differences in bamboo cell walls would be interesting from a fundamental science perspective, as well as in indicating preferential heat pathways.

In this study, we employ scanning thermal microscopy (SThM) to image and map thermal conductivity variations across the bamboo ultrastructure, and relate this to its elegant anatomical organisation. Previous similar studies using scanning thermal microscopy on wood have been fruitful in revealing ultrastructural information (e.g. orientation of cellulose microfibrils in different cell wall layers)^15^, monitoring adhesive penetration at a bond line^16,17^, and assessing the effects of carbonisation between 200 and 600 °C on wood microstructure (e.g. wall thickness) and composition^18^. As far as we know, scanning thermal microscopy has not been used in bamboo research before. We further conclude that scanning thermal microscopy can be a very useful technique in plant science research, including for phenotyping, or even exploring the role of fire regimes and thermal resistance as evolutionary pressures in plant traits.

## Experimental Methods

### Materials

3–5 year old raw Moso bamboo (*Phyllostachys pubescens*) was obtained in whole culm form from China (supplied by UK Bamboo Supplies Limited). The bamboo culms were air-dried and sun-bleached for three weeks upon harvesting, reaching an equilibrium moisture content of 10%. As a reference material, Norway spruce (*Picea abies*) was obtained from BSW Timber Ltd (UK). The spruce wood was cut from flat-sawn, kiln-dried timber with an equilibrium moisture content of 12%.

### Specimen preparation

**Figure 2:**
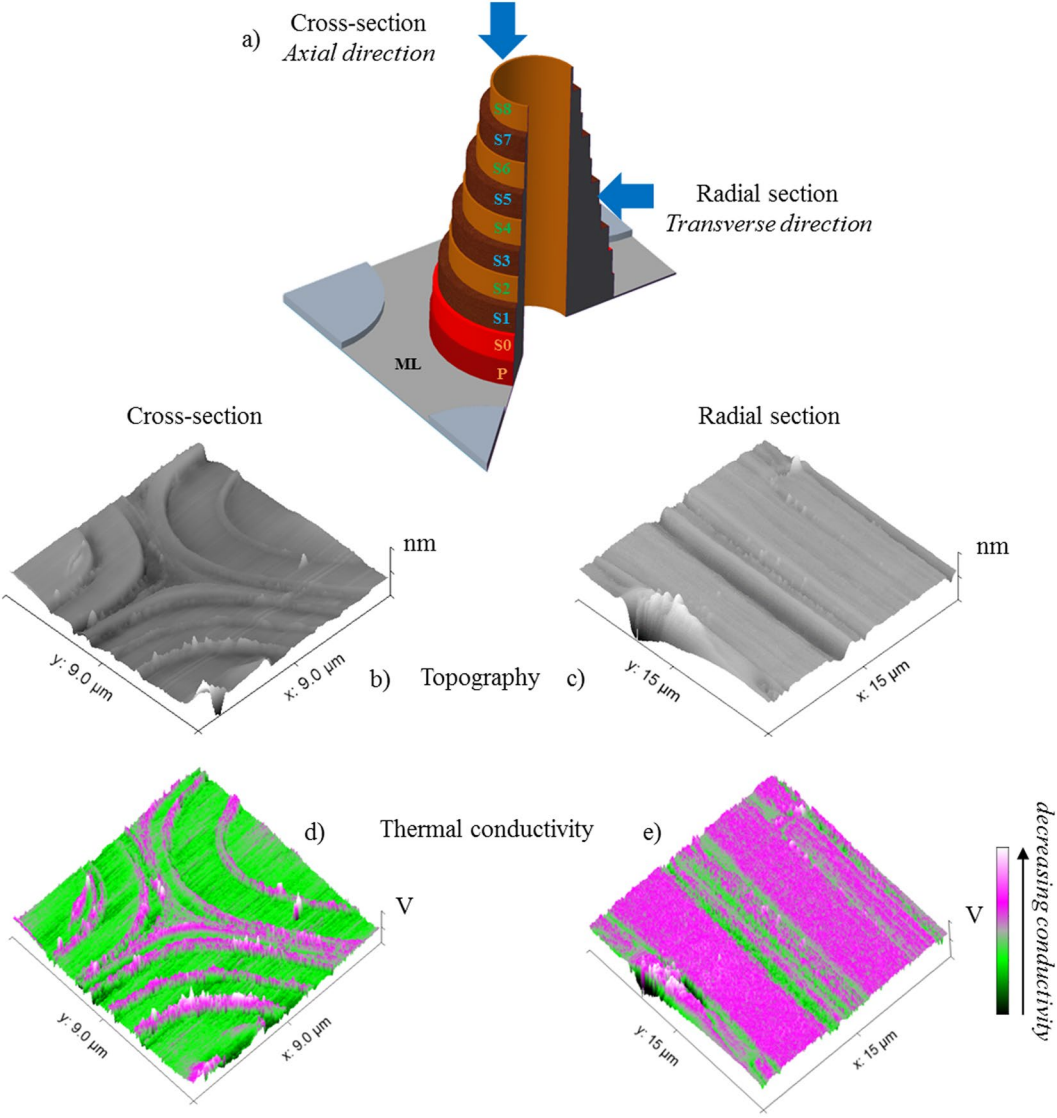
Our setup (**a**) enabled simultaneous acquisition of conventional AFM topography micrographs (**b**,**c**) alongside SThM thermal images (**d**,**e**). Micrographs were captured at cross (**a**,**b**,**d**) and radial (**a**,**c**,**e**) sections of bamboo, as shown, to image thermal conductivity differences between cell wall layers in the longitudinal and transverse directions. In general, higher output voltage [V] implies lower thermal conductivity [W/m·K].

To measure thermal conductivity in the longitudinal (along the stem axis) and transverse directions (perpendicular to the stem axis), cross and radial sections of length 30 mm, width 10 mm and thickness 2–8 mm, were prepared using a sharp razor blade (Fig. 2a). Only inter-nodal regions of the bamboo culm were selected. These sections were impregnated with low viscosity AGAR epoxy resin (AGAR Scientific Ltd., UK) by means of alternating vacuum-pressure treatment. These sections were then glued to 15 mm metal specimen discs for observation in scanning probe microscopy. Local roughness of the sample surface can produce artefacts in the thermal image due to an increase in the tip-sample contact area^15,19^, hence flat surfaces are preferred. To obtain smooth sample surfaces 100 nm thick slices were taken using a Leica Ultracut-R ultramicrotome equipped with a Diatome Histo diamond knife. A more detailed methodology for specimen preparation can be found in^15,16^.

### Scanning thermal microscopy (SThM)

SThM measurements were carried out using a Bruker Dimension Icon Atomic Force Microscope (Bruker, USA) equipped with a SThM-VITA module and a standard Bruker SThM probe (VITA-DM-GLA 1, nominal tip radius  <100 nm, contact mode) and regulated by NanoScope V controller. Scan rate (0.2 Hz) was adapted to scan size (6 to 10 µm squares). From each scan, topographical data and thermal data were simultaneously recorded (Fig. 2b–e). Topographical scans of the surfaces reveal that the roughness root mean squared parameter is typically between 5–30 nm. Given that the nominal tip radius of the probe is <100 nm, the roughness effect on thermal data should be minimal.

**Figure 3:**
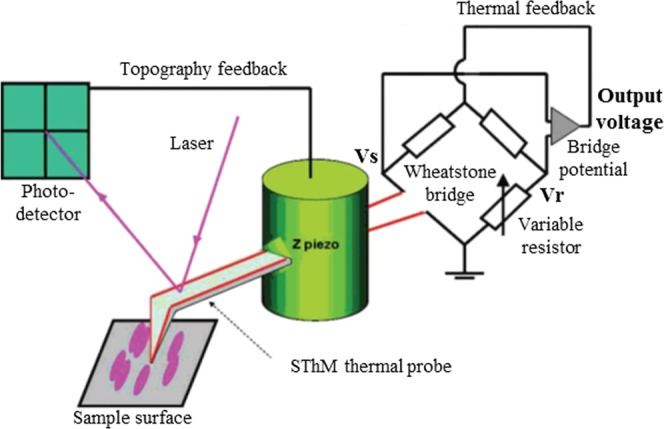
Schematic of the SThM system (adapted from^16^).

Conductivity contrast mode was employed for this series of experiments in which the probe serves as a resistive heater (Fig. 3). When an initial output voltage of 1 V is applied to the SThM thermal probe, the probe temperature elevates above the specimen temperature. Upon contact with the specimen, the probe temperature drops. Some areas of the sample surface will conduct heat less and consequently the probe will be hotter in those areas. Through a Wheatstone bride based feedback mechanism, thermal changes in the probe are measured while the probe temperature is restored to its original value. The energy [W] required to maintain the probe at the initial set temperature [K] represents the local thermal conductivity. Hence, a hot probe will result in higher resistance and higher output voltage of the Wheatstone bridge, and therefore, in general, a higher output voltage [V] implies lower thermal conductivity [W/m·K]. The employed technique allows relative comparison, not absolute measurement, of thermal conductivity.

All measurements were performed with the same probe to prevent influences from varying tip radii, and applied pressure on the sample was kept low to minimize tip wear. Moreover, given the sensitivity of thermal properties of plant-based materials on moisture content^20^, the equilibrated (to ambient conditions) bamboo specimen were tested in a temperature (23 °C) and humidity (55% relative humidity) regulated room over two days. To prevent drift in measurements, control measurements were performed at the beginning and end of scan sessions. Before all measurements, the *Vs-Vr* signal (voltage through the sample probe – voltage through the variable resistor) was set to zero by adjusting the variable resistor (Fig. 3). To optimise scanning parameters, preliminary tests on early and late wood regions of Norway spruce were conducted (results not shown) and verified with results from previous studies^15^. Gwyddion (http://gwyddion.net/qspm/) was used for SThM micrograph data processing.

## Results and Discussion

The bamboo microstructure comprises two principal cell types: high aspect ratio fibre cells in vascular tissue, and brick-shaped parenchyma cells in ground tissue (Fig. 1c). SThM measurements were obtained for these two cell types in cross and radial sections.

**Figure 4:**
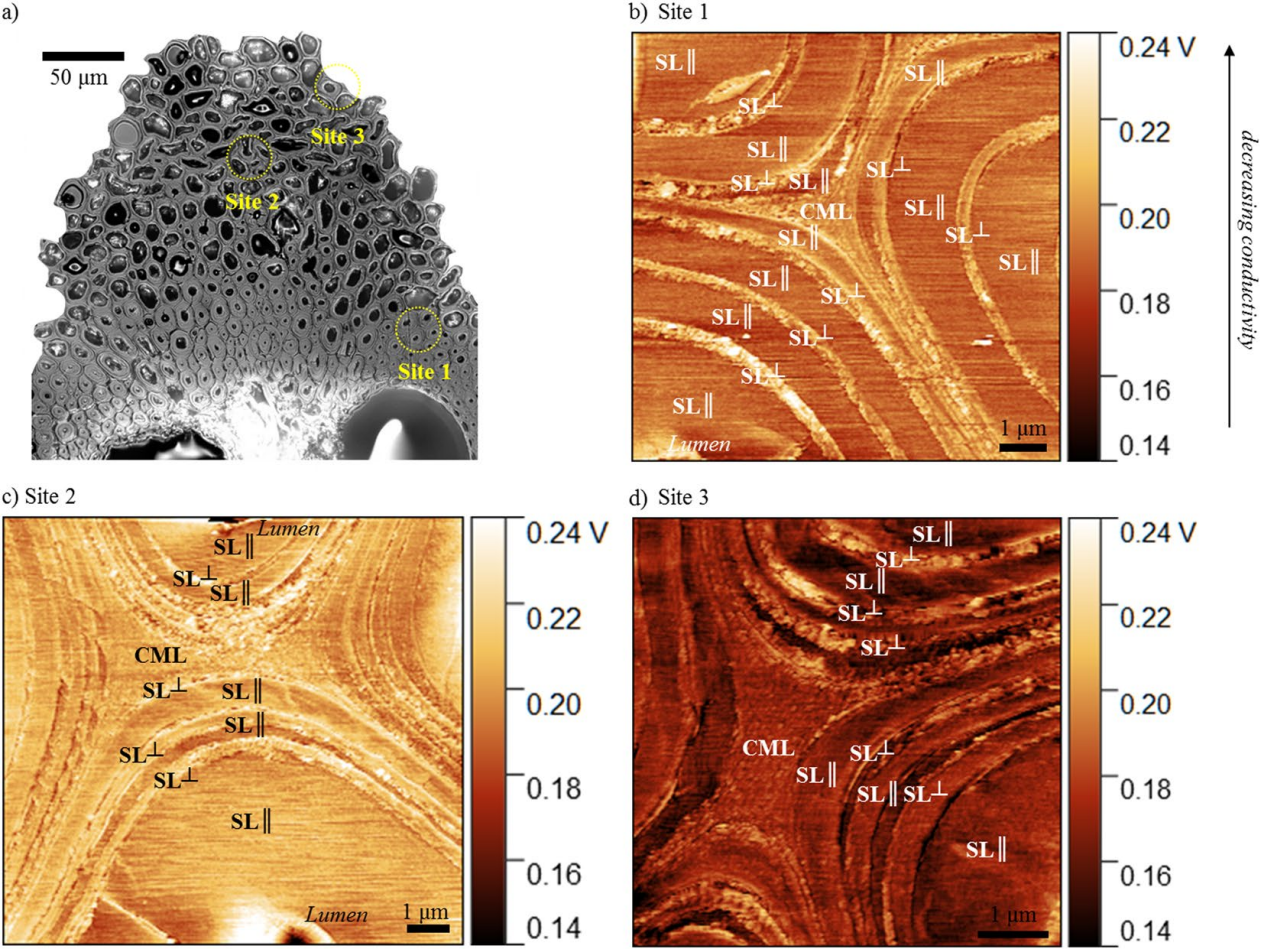
Scanning thermal micrographs depicting axial thermal conductivity variations in cross-sections of bamboo fibre cell wall layers. Alternating layers of thick SL||and thin SL^⊥^ secondary cell walls are observed with higher and lower conductivity, respectively. Micrographs were taken at three different sites (**b**–**d**) on a single vascular bundle of bamboo (**a**). In general, higher output voltage implies lower thermal conductivity.

Topographical scans on cross-sections of vascular tissue revealed the highly multi-lamellar structure of bamboo fibre cell walls, with up to ten layers (Fig. 4b–d). The number of cell wall layers is related to plant maturity^8,21^. Thermal scanning in conductivity contrast mode shed further light on the fibre cell ultrastructure. It was not possible to discern the middle lamella (ML), primary (PL) and the first layer of the secondary (S0) cell wall, indicating they have similar thermal conductivity, or perhaps the probe is too coarse for the thin P and S0 layers. Here, these three combined are referred to as the compound middle lamella (CML). More fascinatingly, in Fig. 4 we observe a repeated and regular alteration of thick and thin secondary cell wall lamellae: thin lamellae (S2, S4, S6…) appear as bright bands, denoting lower conductivity, and the thick lamellae (S3, S5, S7…) appear as dark bands, denoting higher conductivity. The conductivity of the CML was comparable to that of the thinner lamellae.

Each cell wall layer has cellulose microfibrils oriented at various angles to the fibre axis (Fig. 1f), surrounded by an amorphous matrix of hemicelluloses and lignins; the middle lamella joining the adjacent cells is rich in amorphous pectins. The oriented and semi-crystalline cellulose microfibrils exhibit anisotropy in thermal conductivity, with high conductivity along the cellulose microfibrils (*ca* 1 W/m·K^20^), and substantially lower conductivity perpendicular to the microfibrils (*ca* 0.25 W/m·K^20^). The other amorphous and isotropic-behaving cell wall polysaccharides, such as hemicelluloses, lignins and pectins, have comparable conductivity to each other (*ca* 0.35 W/m·K^20^), and similar to conductivity perpendicular to cellulose chains.

Our analysis offers further evidence to the literature-described secondary cell wall architecture of bamboo fibres^7–9,22,23^. Cellulose microfibrils in the thick secondary cell wall lamellae (SL||, dark bands depicting high conductivity) are oriented almost parallel to the fibre cell axis (10–20°), while microfibrils in the thin lamellae (SL^⊥^, bright bands depicting high conductivity) are oriented almost perpendicular to the cell axis (85–90°). In addition, the thin lamellae are more lignified and have higher xylan hemicellulose content than the thick lamellae^7^. Consequently, SL|| cell wall layers have higher thermal conductivity in the fibre direction in comparison to the SL^⊥^ cell wall layers. The compound middle lamella (CML) has a thermal conductivity that is similar to SL^⊥^ cell wall layers.

**Figure 5:**
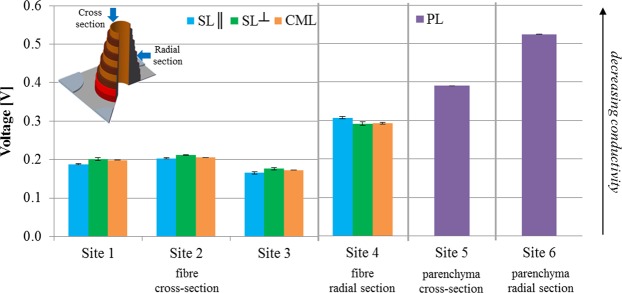
Relative comparison of the thermal conductivity of the different cell wall layers in two cell types: fibre cells (SL||, SL^⊥^, CML) and parenchyma cells (PL). Axial and transverse thermal conductivity, obtained from cross-sections and radial sections, are presented. For this data analysis, area-averaged conductivities of the relevant cell wall regions were obtained from the SThM micrographs in Figs 4 and 6.

Measurements were taken across at least three different sites (Fig. 4b–d, Fig. 5). We observed that while there was some variation in the measured conductivity values across the three sites, which ranged between 0.16–0.21 V, there was a definite trend in axial thermal conductivity of the different fibre cell wall layers: k_SL||_ > k_CML_ ≥ k_SL_⊥. Alongside natural variation in biochemical composition and ultrastructure of bamboo, differences in sample surface roughness and tip-sample contact area, and changing sample temperature over the duration of the experiment are possible sources of observed differences. Notably, differences in fibre cell wall layer thermal conductivity is far larger between anatomical directions (i.e. axial vs transverse conductivity) than that between fibre cell wall layers in one anatomical direction (Fig. 5).

**Figure 6:**
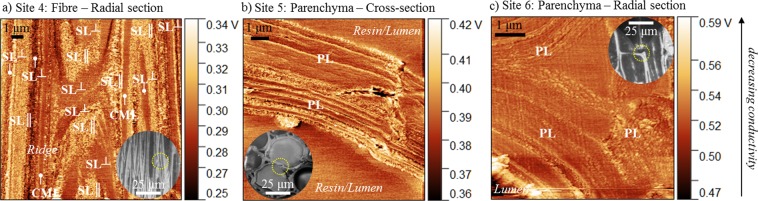
Scanning thermal micrographs taken at (**a**) radial section of a fibre, and (**b**) cross-section and (**c**) radial section of parenchyma cells. In general, higher output voltage implies lower thermal conductivity. Inset images present zoomed-out optical micrographs of the region of interest.

Imaging radial sections of the fibre cells illustrated the anisotropic thermal behaviour of cellulose microfibrils. In particular, transverse thermal conductivity of the SL^⊥^ layers was comparable to the CML, and higher than that of SL|| layers (Figs 5, 6a). However, for all the fibre cell layers, transverse thermal conductivities (0.28–0.31 V) were substantially lower than axial thermal conductivities (0.16–0.21 V). This is understandable as in the cross-section all cellulose microfibrils will be predominantly perpendicular to the section image given their helically-wound nature. Moreover, the heat penetration depth of our measurements is around tens of micrometers^15^, and therefore our measurements will be influenced by not only surface but also sub-surface cellular ultrastructure.

Topography and SThM micrographs of parenchyma cells revealed multiple thin primary cell walls layers (Fig. 6b), possibly due to each layer having a different lignin content^24^. However, fine vertical grooves at the interface of the cell wall layers may have led to scanning artefacts in the thermal image in the form of bright lines (e.g. Fig. 6b). As part of image data processing, these bright lines were not included in the area-average estimation of the thermal conductivity of the parenchyma primary cell wall. As observed in Fig. 5, the parenchyma primary cell wall had higher axial thermal conductivity (0.39 V) than transverse thermal conductivity (0.53 V), likely because of the helical orientation of cellulose microfibrils at ca 30–40° to the cell axis^8,11^. We also found that the parenchyma primary cell wall layers had a notably lower thermal conductivity than all the fibre cell wall layers (Fig. 5). This is attributed to the substantially lower cellulose content and lower cellulose crystallinity in parenchyma cells in comparison to fibre cells^8,11^.

From our scanning probe microscopy analysis (Fig. 5), we can also deduce that at the tissue scale, fibre cells in the scattered vascular tissue would offer preferential pathways for heat transport due to their higher conductivities in both anatomical directions, in comparison to parenchymatic cells in ground tissue. In addition, the transverse orientation offers more resistance to heat flow.

However, we do note that our study only characterised inter-node regions of bamboo. At the node, complex entanglement and quasi-isotropic circumferential orientation of fibre cells^25^ may lead to different heat pathways. Indeed, periodically occurring nodes along the length of a bamboo culm may diffuse and hinder the heat flow path. Notably, much of bamboo material research in literature has focussed on properties of inter-nodal regions^7,10,11,22^, and yet it is clear that the nodal region is also of scientific and industrial interest. For instance, the important biomechanical role of the node structure in preventing buckling failure of the culm^25–27^, and its complex microstructure to facilitate vascular transfer has been reported^28^. Moreover, it is well-known that across a large piece of bamboo with multiple nodal and inter-nodal regions, structural failure initiates preferentially from the nodes^26^, which in turn has implications for the design of engineered bamboo products for the building and construction sector. Hence, a more detailed study illuminating structure-property relations in the nodal regions would be interesting.

## Conclusions

This study employed scanning thermal microscopy to explore thermal conductivity variations between bamboo cells in two anatomical directions. Parenchymatic ground tissue, in general, has a substantially lower thermal conductivity than fibre vascular tissue. This is likely due to lower proportion of aligned crystalline cellulose microfibrils, and higher proportion of amorphous polymers in parenchyma cells. Furthermore, overall thermal conductivity of fibres and parenchyma is higher in the fibre axis than in the transverse direction, due to the higher longitudinal thermal conductivity of cellulose microfibrils. We also observe conductivity variations between fibre cell wall layers. Multi-lamellar bamboo fibre cells are composed of a regular alternation of broad and narrow secondary cell wall lamellae, wherein the broad lamellae (SL||) have cellulose microfibrils oriented almost parallel to the fibre cell axis, while microfibrils in the narrow lamellae (SL^⊥^) are oriented almost perpendicular to the fibre cell axis. Consequently, SL|| have higher conductivity in the fibre direction, and lower conductivity in the transverse directions, in comparison to SL^⊥^. The compound middle lamella (CML) has a thermal conductivity that is similar to SL^⊥^.

While quantitative estimations of thermal conductivities of bamboo cell wall layers were not possible, these observations, combined with detailed information of volumetric composition of different tissues (e.g. by^10^) can be used to estimate thermal conductivities of the different cell wall layers and types.

In general, scanning thermal microscopy is proposed to be a useful technique for natural materials, including for plant phenotyping studies, and anatomical observations of cell wall structure (e.g. layer growth and cellulose fibril orientation).

## Data Availability

The datasets supporting this article can be obtained upon reasonable request through the corresponding author.
